# Peripheral Lipopolyssacharide Rapidly Silences REM-Active LH^GABA^ Neurons

**DOI:** 10.3389/fnbeh.2021.649428

**Published:** 2021-02-25

**Authors:** Jeremy C. Borniger, Luis de Lecea

**Affiliations:** ^1^Cold Spring Harbor Laboratory, Cold Spring Harbor, NY, United States; ^2^Stanford University School of Medicine, Stanford, CA, United States

**Keywords:** lipopolysaccharides, *Escherichia coli*, sleep, lateral hypothalamus, fiber photometry, GABA, VGAT

## Abstract

Immune factors (e.g., cytokines, chemokines) can alter the activity of neuronal circuits to promote “sickness behavior,” a suite of adaptive actions that organisms exhibit in response to infection/injury in order to maximize their chances of recovery (i.e., return to homeostasis). This includes drastic alterations in sleep/wake states, locomotor activity, and food intake, among other behaviors. Despite the ample evidence highlighting interactions between the brain and systemic immunity, studies on how immune challenges alter the activity of genetically defined cell populations controlling arousal states are scarce. As the lateral hypothalamus (LH) serves a major integrative function in behavioral arousal, food intake, and monitoring and responding to changes in systemic physiology, we investigated how GABAergic neurons within this brain region alter their activity across normal sleep/wake states and in response to a peripheral immune challenge with bacterial endotoxin [lipopolysaccharides (LPS)]. Using fiber photometry (GCaMP6s Ca^2+^ signal) in tandem with electroencephalogram (EEG)/EMG recordings to determine arousal states, we observed that population activity of GABAergic neurons in the lateral hypothalamus (LH^GABA^) is highest during rapid-eye-movement sleep (REM), and this activity changes drastically across spontaneous arousal state transitions, with the lowest activity observed during non-REM sleep. Upon intraperitoneal LPS challenge, LH^GABA^ neurons rapidly decrease their activity in tandem with elimination of REM sleep behavior (characteristic of cytokine-induced sickness). Together, these data suggest that peripheral immune challenges can rapidly (in < 40 min) alter subcortical neuronal circuits controlling arousal states. Additionally, we demonstrate that fiber photometry offers a sensitive and cell-type specific tool that can be applied to study the neuronal substrates of sickness behavior.

## Introduction

Sickness behavior is characterized by acute and protracted changes in sleep/wake states, appetitive, sexual, and social behavior, among other changes (Dantzer and Kelley, 2007; Dantzer et al., 2008; Myers, 2008). This suite of adaptive behaviors is largely conserved across the phylogenetic tree, including in humans (Shattuck and Muehlenbein, 2015). Changes in behavior in response to infection/injury can be attributed to neuroimmune crosstalk among peripheral and central cytokines, endothelial cells, glia, and neurons (Henry et al., 2008; Jin et al., 2016). In response to acute inflammatory challenges, such as bacterial endotoxin [lipopolysaccharides (LPS)] exposure, mice increase their time spent in non-rapid-eye-movement (NREM) sleep at the expense of rapid-eye-movement sleep (REM) sleep, with corresponding decreases in wakefulness. These inflammatory signals in the periphery are partially transduced to the brain *via* vagal afferent nerve fibers (Opp, 2005; Zielinski et al., 2013; Borniger et al., 2017). During extended periods of immune activation (e.g., in neurodegenerative disease), sleep becomes fragmented and is accompanied by protracted insomnia (Toth and Krueger, 1988; Dauvilliers, 2007; Irwin and Opp, 2017). The biological substrates that drive this phenomenon remain to be fully identified and characterized, although myeloid cell (microglia/macrophage) interactions with neurons seem to be predominant drivers of infection-associated sleep disruption (Toth and Hughes, 2004). Significant progress has been made in recent years in identifying neural ensembles underlying inflammation-associated changes in feeding behavior (Chaskiel et al., 2016, 2019; Liu et al., 2016; Wang et al., 2019). However, additional work is required to understand how neurons controlling feeding, arousal, and motivational states integrate various inflammatory inputs to induce changes in overt behavior.

The first experimental evidence for humoral (and potentially immune) regulation of sleep/wake states came over a century ago from the Japanese physiologist Kuniomi Ishimori, who demonstrated that cerebrospinal fluid (CSF) from sleep-deprived dogs powerfully induced sleep in naïve recipient dogs (Kubota, 1989; Krueger et al., 2001). Subsequent studies from Fencl et al. (1971) confirmed this finding by injecting CSF from sleep-deprived goats into recipient rats and observed marked increases in sleep. Pappenheimer, Krueger and colleagues subsequently followed up on these findings and termed the mysterious sleep-inducing substance in CSF “Factor S.” Factor S was later determined to be muramyl peptide, a component of bacterial cell walls. Injections of low doses of muramyl peptide or another bacterial cell wall component, LPS, powerfully induced slow wave sleep (SWS) with high amplitude delta (δ) waves (0.5–4 Hz) in the electroencephalogram (EEG) of cats (Krueger et al., 1982, 1984, 1986; Krueger, 1985; Cady et al., 1989). Why would a piece of bacterial cell walls have any influence on mammalian sleep? The evidence thus far supports the hypothesis that the immune response elicited by a bacterial infection indirectly regulates the activity of neuronal structures that control sleep/wake states to putatively overcome the infection/injury and return to homeostasis. In short, the immune system engages sleep circuitry in order to promote rest and recovery.

The hypothalamus represents a key biological substrate underlying the expression of sickness behavior. It controls virtually all homeostatic processes that become dysregulated upon infection/injury, including sleep/wake states, food intake, sexual/social behavior, body temperature, and stress responses, among others (Saper and Lowell, 2014; Bonnavion et al., 2016; Scammell et al., 2017). GABAergic neurons within the lateral division of the hypothalamus (LH^GABA^) represent a heterogenous population of cells that differentially contribute to sleep/wake control. Indeed, single cell RNA sequencing (scRNA-seq) demonstrated that at least 15 sub-populations of GABAergic neurons exist within the lateral hypothalamus (LH) alone (Mickelsen et al., 2019). Major sub-populations include those that express the long-form leptin receptor (LepRb), neurotensin (NTS), or cocaine and amphetamine related transcript (CART) (Bonnavion et al., 2015, 2016; Mickelsen et al., 2019). Whether these subpopulations play similar or differing roles in sleep/wake control is an active area of investigation, but collectively this GABAergic population seems to promote rapid arousal out of NREM, but not REM sleep (Herrera et al., 2016; Venner et al., 2016). Early single-unit electrophysiological studies suggested that LH^GABA^ neurons are active during sleep, with a large proportion active during REM states compared to NREM sleep and wakefulness (Hassani et al., 2010). More recent work demonstrated that approximately 34% of LH^GABA^ neurons are maximally active during REM sleep, and function to reinforce and stabilize hypothalamic representations of feeding behavior (Oesch et al., 2020). Additionally, early studies suggested that LH^GABA^ neurons are preferentially responsive to the inflammatory cytokine IL-1β(De et al., 2002), suggesting that they may play a role in regulating sickness responses governed by the hypothalamus. Further, work has demonstrated that sleep deprivation enhances mRNA expression of a bacterial peptidoglycan recognition protein (PGRP), suggesting a link between bacteria, the hypothalamus, and sleep (Rehman et al., 2001). We aimed to build upon this work by examining population wide LH^GABA^ Ca^2+^ activity during spontaneous arousal state transitions and in response to peripheral endotoxin (LPS) administration. We hypothesized that LH^GABA^ neurons alter their activity across discrete brain states and predicted that we would observe high Ca^2+^ activity during REM sleep, consistent with prior observations. Because of the strong REM-suppressive actions of bacterial endotoxin, we reasoned that LH^GABA^ neurons would drastically alter their activity across arousal states during the inflammatory response.

## Materials and Methods

### Mice

Heterozygous VGAT-IRES-Cre mice (*N* = 5; male, >8 weeks old; JAX # 028862) were used in these experiments, housed with *ad libitum* food and water at room temperature (22 ± 2°C). The dental cement headcap of one mouse became disconnected (and therefore required euthanasia) between initial recordings and subsequent LPS experiments, so *N* = 4 for the LPS data. These mice were bred in-house and maintained on the c57bl6 background for >10 generations prior to use. Cre + mice were used for all experiments, and genotyping was done using tail gDNA and primers targeting Cre sequences (Cre_F: GGGATTGCTTATAACACCCTGTTACG; Cre_R: TATTCGGATCATCAGCTACACCAGAG). All experimental protocols were approved by the Stanford University IACUC prior to beginning these studies (protocol #18787).

### Surgery

Electroencephalogram/EMG electrodes were custom made as in previous work (Carter et al., 2012; Eban-Rothschild et al., 2016, 2020). Mice were deeply anesthetized with ketamine/xylazine (90 mg kg^–1^/10 mg kg^–1^, respectively), and then their head was shaven and cleaned with betadine/EtOH. Mice were then positioned on a stereotaxic frame for surgery and administered prophylactic analgesia (buprenorphine SR; 1 mg kg^–1^; SC). Mice were maintained on a low-power heating blanket during surgery to prevent hypothermia. The skin on the head was cut from the posterior margin of the eyes until the midpoint of the scapulae, and then cleaned using H_2_O_2_ and sterile saline. Three holes were drilled into the skull. Two holes (one at 1.5 mm AP, 1 mm ML; the other on the contralateral hemisphere at −2 mm AP, −1.5 mm ML) were to be used for placement of cortical electrodes for EEG measurement, and the last hole was used for viral injections into the LH (−1.2 mm AP, ± 0.89 mm ML, −5.3 mm DV). A Hamilton syringe (0.36 mm outer diameter; 28 gauge) loaded with 300 nl AAV-DJ-DIO-ef1α-GCaMP6s [titer > 1 × 10 (Dauvilliers, 2007) vg/ml; Stanford Viral Vector Core] was slowly lowered to these coordinates and virus was injected unilaterally at 100 nl/min. Following injection, the syringe was left in place for an additional 10 min to prevent backflow, then the syringe was slowly removed. A 400 μm diameter fiber optic (Doric Lenses; 6 mm in length) was slowly lowered to just above the virus injection coordinate (−5.2 mm DV) and then cemented into place using Metabond (Parkell, Inc.) and UV-curable dental resin. EMG leads were inserted and secured in the trapezius muscles, and EEG screws were cemented into place. After fiber and EEG/EMG placement, any surgical openings were sutured closed, and then the mice were given supplemental warmth (heating pad) and hydration (0.4 ml saline, SC) until mobile. Mice were allowed to recover for at least 3 weeks before starting experiments (to allow for adequate transgene expression). Before starting recordings, mice were separated and allowed to acclimate to a specialized (open top) sleep recording chamber, EEG/EMG tethers, and the fiber optic patchcord for at least 3 days.

### LPS Administration

Mice were administered 0.5 mg kg^–1^ LPS (serotype O111:B4; Sigma-Aldrich) in sterile saline (intraperitoneal; IP) as in prior work (Borniger et al., 2017) at zeitgeber time (ZT) 4, when sleep pressure has mostly abated. Forty minutes later, photometry and EEG signals were collected for an additional 40 min to monitor LH^GABA^ Ca^2+^ activity in tandem with sleep/wake states. For control/baseline recordings, mice were administered sterile saline vehicle alone (200 μl; IP). We chose to record 40 min after IP injections to reduce the influence stress had on the neural/sleep phenotype.

### Fiber Photometry

Fiber photometry is an increasingly common method within the neuroscience community by which an optic fiber is placed near a brain region of interest and light is delivered and collected from the fiber tip. Cells within the target area are engineered (e.g., *via* viral transfection) to express a fluorescent protein (e.g., GCaMP6s) that, upon light excitation, transduces Ca^2+^ activity (a proxy of neural activation) into a high-temporal resolution fluorescent signal that is collected by the optic fiber, converted into a voltage signal, and subsequently analyzed. Because of the small and lightweight design of optic fibers, these recordings can be done in freely moving mice, as in the present study. In line with this, fiber photometry experiments were conducted as previously (Eban-Rothschild et al., 2016; Giardino et al., 2018; Li et al., 2020). In brief, we modulated blue light from a 470-nm excitation LED (M470F3, ThorLabs, NJ, United States) at 211 Hz, using a custom MATLAB program (MathWorks, Natick, MA, United States) and a multifunction data acquisition device (NI USB-6259, National Instruments, Austin, TX, United States). Blue light was passed through a GFP excitation filter (MF469-35, ThorLabs), bounced off a dichroic mirror (MD498, ThorLabs), and then coupled using a fiber collimation package (F240FC-A, ThorLabs) into a low-fluorescence patch cord (400 μm, 0.48 NA; Doric Lenses) *via* a zirconia sleeve (Doric). GCaMP6s fluorescence was collected through the excitation patch cord, where it traveled through a GFP emission filter (MF525-39, ThorLabs), and focused onto a photodetector with a lens (Model 2151, Newport, Irvine, CA, United States; LA1540-A, ThorLabs). The signal was relayed to a lock-in amplifier (30-ms time constant, Model SR830, Stanford Research Systems, Sunnyvale, CA, United States) that was synchronized to 211 Hz. Amplified signals were collected at 1 kHz using custom MATLAB code and a multifunction data acquisition device (National Instruments).

### EEG/EMG

Sleep/wake states were assessed *via* EEG/EMG. These biopotentials were sampled at 256 Hz with VitalRecorder (Kissei Comtec Co.) software, and then passed through an amplifier (Grass Instruments). Raw EEG/EMG voltage data were exported (.txt) into MATLAB (MathWorks, Natick, MA, United States) and analyzed using custom scripts [as in Li et al. (2020)]. Wake was defined as low amplitude, high frequency EEG oscillations with prominent EMG signal. NREM sleep was characterized by large amplitude, low frequency delta oscillations in the EEG, with little or no EMG signal. REM sleep was identified based on a predominant theta (θ) component of the EEG, with no EMG activity reflecting complete motor atonia. Behavioral states (wake, NREM, or REM sleep) were assessed blind to the time-locked photometry signal or experimental manipulation (LPS or saline).

### Histology

After completion of experiments, mice were deeply anesthetized using ketamine/xylazine and then perfused (transcardial) with ice-cold 1x PBS followed by 4% paraformaldehyde (PFA). Brains were dissected and post-fixed in 4% PFA at 4°C overnight, followed by cryoprotection in 30% sucrose for 2–3 days until sunk. Serial sections were cut on a cryostat (Leica Microsystems) at 30 μm and placed into 24-well plates containing 1x PBS + 0.1% NaN_3_, covered with aluminum foil, and maintained at 4°C until histology/immunohistochemistry. Validation of GCaMP6s viral transduction and fiber placement was confirmed *via* confocal microscopy (Excitation λ: 488 nm; Emission λ: 509 nm; LSM-710; Zeiss).

### Data Analysis and Statistics

Electroencephalogram/EMG data and time-locked photometry data were analyzed using custom MATLAB code. In brief, photometry data was corrected for linear or exponential decay in the trace [as in Eban-Rothschild et al. (2016) and Eban-Rothschild et al. (2020)], which occurred in a minority of recordings. Photometry data was down sampled to 256 Hz in order to match the EEG signal sampling rate. Data from each arousal state transition (i.e., wake-to-NREM, NREM-to-wake, NREM-to-REM, and REM-to-Wake) were analyzed for the mean and max fractional fluorescence change (dF/F) in each state, and then time series were generated for 10 s preceding and 10 s following each transition. Differences among experimental groups was assessed using one-way ANOVA, followed by Tukey’s HSD *post hoc* test. For analysis of GCaMP6s spectral data, 2-way repeated measures ANOVAs were used with treatment and frequency bin as independent factors, followed by Šidák’s multiple comparisons *post hoc* test.

## Results

### LH^GABA^ Neurons Are Predominantly REM-Active

**FIGURE 1:**
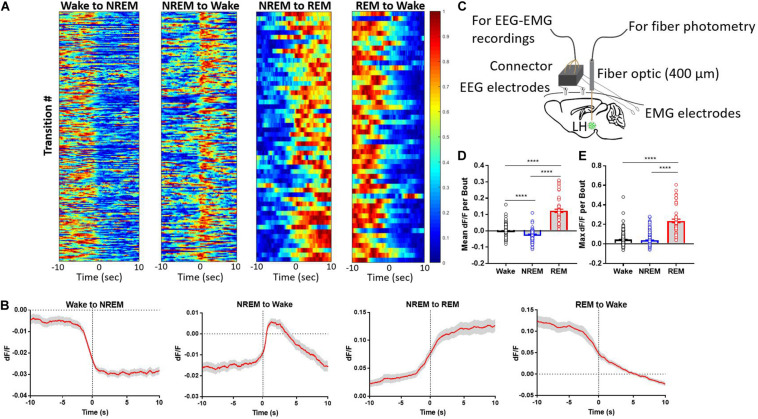
Lateral hypothalamic GABAergic neurons are wake- and REM sleep-active. **(A)** Heatmaps showing GCaMP6s signal across arousal state transitions (Wake-to-NREM (256 transitions), NREM-to-Wake (*n* = 208 transitions), NREM-to-REM (*n* = 60 transitions), and REM-to-Wake (*n* = 57 transitions), with transition number on the y-axis and time on the x-axis; GCaMP6s signal is reflected in the colormap. **(B)** The same data as in panel **(A)** but collapsed into the average signal time series across arousal state transitions. **(C)** Experimental design describing the set up for simultaneous recording of EEG/EMG + fiber photometry signal from freely moving mice. **(D)** mean dF/F of LH^GABA^ neuronal activity per bout of each arousal state (One-way ANOVA *F*_2, 591_ = 438.1; *p* < 0.0001); (Tukey’s *post hoc* wake vs. NREM: *q* = 10.72, adjusted *p* < 0.0001; wake vs. REM: *q* = 35.58, adjusted *p* < 0.0001; NREM vs. REM: *q* = 41.84, adjusted *p* < 0.0001). **(E)** max dF/F of LH^GABA^ neuronal activity per bout of each arousal state (One-way ANOVA *F*_2, 591_ = 212.9; *p* < 0.0001); (Tukey’s *post hoc* wake vs. REM: *q* = 27.29, adjusted *p* < 0.0001; NREM vs. REM: *q* = 28.45, *p* < 0.0001). Error bars represent S.E.M., *N* = 5 mice, ****p < 0.0001, One-way ANOVA followed by Tukey’s HSD *post hoc* test).

We monitored LH^GABA^ Ca^2+^ activity during natural arousal state transitions. These neurons exhibited marked changes in GCaMP fluorescence across arousal states, with the highest mean fractional fluorescence change (ΔF/F or dF/F) during REM sleep compared to wakefulness or NREM sleep (Figure 1). Additionally, these neurons were intermediately active during wakefulness compared to REM and NREM sleep, respectively (Figure 1D; One-way ANOVA *F*_2, 591_ = 438.1; *p* < 0.0001; Tukey’s *post hoc* wake vs. NREM: *q* = 10.72, adjusted *p* < 0.0001; wake vs. REM: *q* = 35.58, adjusted *p* < 0.0001; NREM vs. REM: *q* = 41.84, adjusted *p* < 0.0001). In contrast, NREM sleep and wakefulness showed similar maximal ΔF/F values, but REM sleep reached upward of 20–30% ΔF/F state (Figure 1E; One-way ANOVA *F*_2, 591_ = 212.9; *p* < 0.0001); (Tukey’s *post hoc* wake vs. REM: *q* = 27.29, adjusted *p* < 0.0001; NREM vs. REM: *q* = 28.45, *p* < 0.0001). These results indicate that LH^GABA^ Ca^2+^ activity peaks during REM sleep, suggesting that this heterogenous subset of neurons likely plays a role in REM sleep-related phenomena.

### Endotoxin Rapidly Silences LH^GABA^ Neurons

**FIGURE 2:**
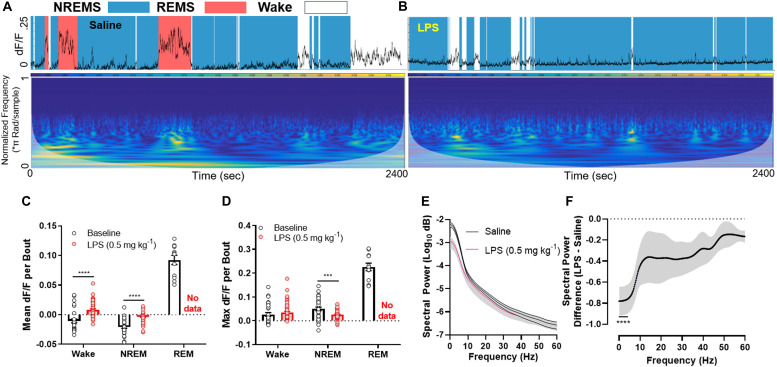
LPS rapidly silences LH^GABA^ neurons. **(A)** Representative GCaMP6s trace and scaleogram from a mouse 40 min following a saline injection. The trace is shaded by the arousal state the animal was in at the time (based on EEG/EMG recordings), where blue = NREM sleep, red = REM sleep, and white = wakefulness. The colormap for the scaleogram represents the power. **(B)** representative GCaMP6s trace and scaleogram from the same mouse 40 min following a 0.5 mg kg^– 1^ LPS IP injection. Note the absence of high amplitude GCaMP signal in tandem with loss of REM sleep. **(C)** quantification of all data in the experimental set showing the mean dF/F per bout between baseline and LPS groups (wakefulness: *t*-ratio = 8.239, adjusted *p* < 0.0001; NREM sleep: *t*-ratio = 6.057, *p* < 0.0001). **(D)** quantification of all data in the experimental set showing the max dF/F per bout between baseline and LPS groups (NREM sleep: *t* = 4.020, *p* < 0.001) (Multiple *t*-tests, Holm–Sidak method). **(E)** Mean spectral power of the GCaMP6s signal among the entire experimental set for saline and LPS treated mice. Note the disparity in power at the lower (<10 Hz) frequencies. **(F)** The same data as in panel **(E)** except the difference is calculated for ease of viewing (2-way RM ANOVA main effect of LPS treatment *F*_1,6_ = 11.85, *p* = 0.0138; main effect of frequency *F*_240, 1440_ = 24.74, *p* < 0.0001; interaction of treatment × frequency *F*_240, 1440_ = 11.44, *p* < 0.0001; individual data points were analyzed using *post hoc* Sidak’s multiple comparisons test (significance denoted by **** where all values are significant until 4.25 Hz on the x-axis). Note a ∼80% decrease in low frequency power in response to LPS treatment. Error bars represent S.E.M., ****p < 0.0001, ***p < 0.001, and **p < 0.01; *N* = 4 mice.

Upon injection of LPS, mice rapidly entered NREM sleep at the expense of REM sleep. This was associated with widespread silencing of LH^GABA^ neurons throughout the entire recording session (Figures 2A–D; mean dF/F per bout between baseline and LPS groups (wakefulness: *t*-ratio = 8.239, adjusted *p* < 0.0001; NREM sleep: *t*-ratio = 6.057, *p* < 0.0001); max dF/F per bout between baseline and LPS groups (NREM sleep: *t* = 4.020, *p* < 0.001) (Multiple *t*-tests, Holm–Sidak method). Upon closer inspection of the data, we noted that the GCaMP6s signal showed reductions primarily in the lower frequency ranges (<10 Hz), where large, coordinated bursts of calcium activity (and putatively neuronal activity) occur (Figures 2E,F; 2-way RM ANOVA main effect of LPS treatment *F*_1,6_ = 11.85, *p* = 0.0138; main effect of frequency *F*_240, 1440_ = 24.74, *p* < 0.0001; interaction of treatment × frequency *F*_240, 1440_ = 11.44, *p* < 0.0001). Šidák’s multiple comparisons *post hoc* tests identified all frequency bins below 4.25 Hz were significantly different between groups (0.25 Hz frequency steps; Figure 2F). There was no compensation in higher frequency ranges, suggesting the effect we observed was due to a reduction in coordinated firing rather than simple desynchronization of the local LH^GABA^ network. These data link inflammation-induced suppression of REM sleep to the silencing of hypothalamic neurons that regulate arousal states.

## Discussion

Our experiments identify LH^GABA^ neurons as putative central sensors of peripheral immune activation. We observed that LH^GABA^ neurons are primarily REM-active, an arousal state powerfully inhibited by acute immune challenge (i.e., with LPS). Prior work demonstrated that these neurons are integrated into arousal circuitry in a unique way. Optogenetic stimulation of LH^GABA^ neurons during NREM, but not REM sleep, rapidly induces wakefulness (Herrera et al., 2016). This aligns with our observations of maximal LH^GABA^ Ca^2+^ activity during REM sleep, which may represent a physiological “ceiling” that further optogenetic stimulation cannot break.

GABAergic neurons in the lateral hypothalamus provide powerful inhibitory control over GABAergic neurons within the thalamic reticular nucleus (TRN). Direct activation of TRN neurons results in diverse electrocortical and behavioral outputs, which are largely dependent on the stimulation frequency and modality (e.g., electrical vs. optical). These effects range from increases in cortical gamma (∼30–150 Hz) (Macdonald et al., 1998), NREM spindles (Halassa et al., 2011; Kim et al., 2012), and EEG slow waves (Kim et al., 2012). A few years ago, Herrera et al. (2016) identified LH^GABA^ neurons as a major source of inhibitory input to TRN^GABA^ neurons, directly linking a feed forward inhibitory circuit to dynamic changes in behavior and cortical activity. This positioned LH^GABA^ neurons as major players in sleep/wake state transitions and overall arousal state. Our data extend these findings and suggest that immune-mediated modulation of this neuronal population may play a role in the regulation of arousal in response to infection/injury, although this remains to be tested empirically. It is relevant that other hypothalamic populations, including the sleep-regulating preoptic neurons, seem to be disposable in mediating the effect of inflammation (muramyl dipeptide) on sleep (Shoham et al., 1989). This lends credence to the idea that discrete cell populations within this brain structure enact a coordinated response to inflammatory signals, rather than a general and non-specific response.

Another major aspect of sickness is a change in the expression of motivated behavior (Lasselin et al., 2017), which is naturally intertwined with arousal. An important characteristic of a motivational state is that it competes with other motivational states for behavioral output. The normal expression of behavior re- quires a hierarchical organization of motivational states that is continuously updated according to the circumstances. When an infection occurs, the sick individual is at a life or death juncture and his/her physiology and behavior must be altered so as to overcome the disease. However, this is a relatively long-term process that needs to make room for more urgent needs when necessary. It is easy to imagine the following [from Dantzer (2001)]: “if a sick person lying in his/her bed hears a fire alarm ringing in his/her house and sees flames and smoke coming out of the basement, he/she should be able to momentarily overcome his/her sickness behavior to escape danger”(Dantzer, 2001). In motivational terms, fear competes with sickness, and fear-motivated behavior takes precedence over sickness behavior. An example of this competition between fear and sickness is provided by the observation that the depressing effects of IL-1β on behavior of mice are more pronounced when experimental animals are tested in the safe surroundings of their home cage than when they are placed into a new environment (Propes and Johnson, 1997; Dantzer, 2001). As LH^GABA^ neurons regulate diverse motivated behaviors, in part *via* their projections to the midbrain dopaminergic system (Nieh et al., 2016), future work should examine how inflammatory signaling alters their activity across various motivational states.

Our findings on LH^GABA^ dynamics during immune challenge are especially relevant given the role these neurons play in appetitive and consummatory behaviors dysregulated during sickness. Seminal studies from the middle of the 20th century revealed that electrical stimulation of the LH (and putatively GABA neurons) promotes voracious feeding and appetitive reward-related behaviors (Hoebel and Teitelbaum, 1962; Margules and Olds, 1962), while lesioning this area elicited aphagia and subsequent emaciation (Anand and Brobeck, 1951; Hoebel, 1965). Subsequent work demonstrated that the actions of the LH on consummatory/appetitive behavior are conserved across evolutionary time and persist in humans (Quaade et al., 1974; Molina-Borja and Gómez-Soutullo, 1989). Stuber and colleagues used cell-type specific tools to bolster these early findings and identified LHGABA neurons as major drivers of feeding and reward behavior (Jennings et al., 2015). Optogenetic stimulation of these neurons (20 Hz) increased the time mice spent in a designated food area, enhanced food consumption, and promoted a preference for the location that was previously paired with food consumption. Reciprocally, photoinhibition of these neurons elicited opposite phenotypes, where mice reduced food consumption and time spent in a location paired with optical inhibition. Appetitive and consummatory behaviors toward food are powerfully suppressed during immune activation (O’Reilly et al., 1988; Langhans et al., 1990), and hypophagia/anorexia is a hallmark of sickness behavior (Dantzer and Kelley, 2007; Pecchi et al., 2009). Our data support the notion that LHGABA neurons are critical regulators of sickness-induced sleep. However, it remains to be determined whether this same cell population links immune activation to reductions in food intake. If true, augmentation of LH^GABA^ activity may ameliorate several aspects of sickness behavior.

Our study has a few key limitations that are worth discussing. We did not measure EEG/EMG and photometry signals during the entire course of LPS-induced sickness (up to 24 h). This was done due to the potential of constant illumination to photobleach the GCaMP reporter, which would prevent appropriate interpretation of the data. Therefore, it remains unclear whether LH^GABA^ neurons recover their normal activity prior to the resolution of overt sickness behavior. Additionally, we cannot make causal inferences from these data, as the contributions of other neuronal populations on LH^GABA^ neuronal activity and sleep/wake states was not eliminated (e.g., hypocretin/orexin neurons) (Bonnavion et al., 2015). Indeed, LPS was shown to powerfully suppress hypocretin/orexin and histaminergic neuronal activity (cFos immunolabeling) during the dark phase in rats, when wakefulness and cellular activity are highest (Gaykema and Goehler, 2009). Comparative studies in mice with intact hypocretin/orexin neurons and those lacking this cellular population (ataxin-3 mice) demonstrated that LPS powerfully inhibited hypothalamic hypocretin expression in both genotypes but was only completely abolished in ataxin-3 mice. LPS also suppressed a potential compensatory increase in histamine decarboxylase-positive cells within the hypothalamus of ataxin-3 mice, which implies the involvement of multiple neuromodulator systems in inflammation-associated sickness (Tanaka et al., 2016). A major caveat that may influence the conclusions that we draw from these data are that bulk photometry recordings cannot delineate whether LPS silenced all LH^GABA^ neurons equally [as there are many subtypes (Mickelsen et al., 2019)], or whether their firing rates were fundamentally unchanged and simply desynchronized in their output. However, spectral analysis of the GCaMP signal (Figure 2) suggests that LPS reduced low frequency/high amplitude calcium waves, without additional increases in higher frequency oscillations, suggesting a net reduction in coordinated neuronal output rather than desynchronization. Subsequent miniscope or single unit electrophysiological studies will be needed to definitively answer these questions. We also did not measure the extent of neuroinflammation induced by the fiber optic surgery itself, which may have influenced the somnogenic and neuromodulatory properties of LPS. In line with this, to provide a comprehensive picture of LH^GABA^ responses to LPS, multiple doses should be used in future studies. Finally, the downstream outputs of these neurons driving sickness behavior was not examined, and the effects of LH^GABA^ neurons may be secondary to a different population within the LH or other nuclei. Viral-mediated tract tracing studies and genetic ablation techniques will help to answer these questions.

## Data Availability Statement

The raw data supporting the conclusions of this article will be made available by the authors, without undue reservation.

## Ethics Statement

The animal study was reviewed and approved by Stanford University IACUC.

## Author Contributions

JB: experimental design, performed experiments, analyzed data, wrote manuscript, and edited manuscript. LL: experimental design, study supervision, supplied critical resources/tools, and edited manuscript. Both authors contributed to the article and approved the submitted version.

## Conflict of Interest

The authors declare that the research was conducted in the absence of any commercial or financial relationships that could be construed as a potential conflict of interest.
